# Playing posture, instrument characteristics, and musculoskeletal pain among adolescent wind instrument players: an exploratory cross-sectional study

**DOI:** 10.1186/s12891-026-10061-2

**Published:** 2026-06-05

**Authors:** Yasuhiro Mitani, Maki Koyanagi, Masaru Notani

**Affiliations:** 1 Department of Rehabilitation Sciences, Faculty of Allied Health, Kansai University of Welfare Sciences, 3-11-1 Asahigaoka, Kashiwara, Osaka 582-0026 Japan; 2 Course of Physiotherapy, Faculty of Health Informatics, Osaka Electro- Communication University, 1130-70 Kiyotaki, Shijonawate, Osaka 575-0063 Japan; 3 Department of Rehabilitation, Gratia Hospital, 6-14-1 Aomadaninishi, Minoo, Osaka 562-8567 Japan

**Keywords:** Playing-related musculoskeletal disorders, Posture, Pain, Low-back pain, Wind instrument players

## Abstract

**Background:**

Playing-related musculoskeletal disorders (PRMDs) in musicians commonly develop around the neck-and-shoulder region and the low back. This study investigated the onset of neck-and-shoulder and low-back pain among wind instrument players in a high school brass band and examined how instrument weight and playing posture relate to the occurrence, severity, and duration of such pain.

**Methods:**

This exploratory cross-sectional study included 120 high school wind instrument players who completed a questionnaire survey regarding their experience with playing-related pain in the neck-and-shoulder region and low back, and pain severity and duration while playing the instrument. The chi-square test was used to determine the possible relationships between the study parameters and playing posture and instrument weight.

**Results:**

A total of 82 students (68.3%) experienced pain in the neck-and-shoulder region, whereas 53 (44.2%) experienced low-back pain. A significantly higher proportion of students who played light instruments reported low-back pain compared with those who played heavy instruments (*p* < 0.05), possibly reflecting sustained unsupported playing postures required for many light instruments. Moreover, a significantly higher proportion of participants with an asymmetrical playing posture reported moderate-to-severe low-back pain (NRS ≥ 4) lasting ≥ 3 months than those with a symmetrical playing posture (*p* < 0.05). Although asymmetrical posture was observed across all instrument groups, these findings suggest that posture-related factors may contribute to the persistence of low-back pain. Instrument weight and playing posture were not significantly associated with pain severity or duration in the neck-and-shoulder region.

**Conclusions:**

This exploratory cross-sectional study found that instrument weight and playing posture were not significantly associated with neck-and-shoulder region pain. However, more students who played light instruments had low-back pain compared to those who played heavy instruments. Moreover, an asymmetrical playing posture was associated with greater severity and longer duration of low-back pain. These results suggest that asymmetrical playing posture may be associated with greater biomechanical demands on the low back during performance, even among light-instrument players, and highlight important implications for the prevention of PRMDs and education for high school wind instrument players.

**Supplementary Information:**

The online version contains supplementary material available at 10.1186/s12891-026-10061-2.

## Background

Playing-related musculoskeletal disorders (PRMDs) are a common problem among musicians, with overuse resulting from prolonged practice and performance identified as a primary causative factor [1–5]. This is particularly evident in the fingers and hands of keyed and stringed instrument players, in whom repetitive motion is intrinsic to playing [4,6].

PRMDs frequently develop not only in the fingers and hands but also in the neck, shoulders, and low back, [1, 7] and these disorders are theorized to be related to instrument-playing posture [8, 9]. Edling et al. [9] investigated this relationship in 47 music teachers and reported that an asymmetrical playing posture may increase the risk of neck, shoulder, and low-back disorders. However, due to several limitations, including a small sample size, inclusion of various instrument types, variation in playing frequency, and a wide participant age range, this relationship could not be definitively confirmed [9]. Notably, the wide age range represents a critical limitation, as factors contributing to PRMDs may differ between adolescents and adults. Of particular public health relevance, low-back pain during adolescence is a known predictor of low-back pain in adulthood [10, 11]. Therefore, identifying factors contributing to PRMDs in adolescent musicians is essential for developing effective preventive strategies. Consequently, investigations should standardize participant characteristics, particularly age, to clarify the relationship between playing posture and PRMDs.

In addition, Mitani et al. [12] measured muscle activity in the cervical paraspinal muscle, the upper fiber of the trapezius, and the lumbar paraspinal muscle while holding the flute, trumpet, and marching euphonium playing postures while standing. The results showed that the activities of these muscles were increased not only with the marching euphonium, which is a heavy instrument, but also with the flute, which has an asymmetrical playing posture. These findings suggest that both playing posture and instrument characteristics may influence physical load on the neck-and-shoulder region and low back and are possibly related to pain. However, instrument weight alone may not adequately represent the biomechanical stress imposed during performance; factors such as trunk posture, forward inclination, sustained static muscle activity, and the method of instrument support—collectively referred to as “functional” or “postural” load—may reflect the physical demands of playing more accurately. Accordingly, investigations on PRMDs need to consider the broader concept of functional load along with instrument weight and playing posture.

Therefore, the present study investigated the onset of pain in the neck-and-shoulder region and low back among wind instrument players in a high school brass band and examined its relationship with instrument weight and playing posture. This study hypothesized that pain in the neck-and-shoulder region and low back would be more prevalent, more severe, and of longer duration among players with asymmetrical playing postures and those playing instruments associated with greater functional or postural load.

## Methods

### Participants

The participants included 120 wind instrument players (13 boys and 107 girls; 55 first-year students and 65 s-year students; mean age: 16.0 ± 0.7 years) who were members of a brass band of a public high school in Osaka prefecture, Japan. Instrument playing history was < 1 year, 1 to < 3 years, and ≥ 3 years in 25, 32, and 63 participants, respectively. The participants practiced 6 days per week, for approximately 2 h per day on weekdays and 6 to 8 h per day on holidays. No marching band was formed out of this brass band. A total of 13 instrument types were included. Instruments weighing < 1.0 kg were classified as “light,” whereas those weighing ≥ 1.0 kg were classified as “heavy.” The 1.0 kg cutoff was determined a priori by consensus among the authors for practical purposes in this exploratory study. This threshold provides an operational distinction between smaller handheld instruments and larger instruments, which typically require additional structural support during performance (e.g., neck straps, stands, or thigh support) (Table 1). The predominance of female participants (107 of 120; 89.2%) reflected the actual demographic composition of the brass band at the school and was inherent to the study setting. Instrument playing history was recorded for descriptive purposes and not included as an explanatory variable in the statistical analyses.

**Table 1:** Instrument types and number of participants with neck-and-shoulder region and low-back pain

	Instrument weight	Playing posture symmetry	Neck-and-shoulder region pain		Low-back pain	
			Yes *n* (%)	No *n* (%)	Yes *n* (%)	No *n* (%)
Flute (*n* = 12)	Light	Asymmetrical	10 (83.3%)	2 (16.7%)	9 (75.0%)	3 (25.0%)
Oboe (*n* = 3)	Light	Symmetrical	0 (0.0%)	3 (100.0%)	1 (33.3%)	2 (66.7%)
Clarinet (*n* = 23)	Light	Symmetrical	15 (65.2%)	8 (34.8%)	13 (56.5%)	10 (43.5%)
Trumpet (*n* = 16)	Light	Symmetrical	12 (75.0%)	4 (25.0%)	7 (43.8%)	9 (56.3%)
Trombone (*n* = 12)	Heavy	Asymmetrical	8 (66.7%)	4 (33.3%)	2 (16.7%)	10 (83.3%)
French horn (*n* = 12)	Heavy	Asymmetrical	7 (58.3%)	5 (41.7%)	4 (33.3%)	8 (66.7%)
Bass clarinet (*n* = 5)	Heavy	Symmetrical	3 (60.0%)	2 (40.0%)	0 (0.0%)	5 (100.0%)
Alto saxophone (*n* = 10)	Heavy	Asymmetrical	7 (70.0%)	3 (30.0%)	4 (40.0%)	6 (60.0%)
Euphonium (*n* = 6)	Heavy	Asymmetrical	5 (83.3%)	1 (16.7%)	4 (66.7%)	2 (33.3%)
Tenor saxophone (*n* = 6)	Heavy	Asymmetrical	2 (33.3%)	4 (66.7%)	3 (50.0%)	3 (50.0%)
Bassoon (*n* = 5)	Heavy	Asymmetrical	5 (100.0%)	0 (0.0%)	3 (60.0%)	2 (40.0%)
Baritone saxophone (*n* = 3)	Heavy	Asymmetrical	3 (100.0%)	0 (0.0%)	1 (33.3%)	2 (66.7%)
Tuba (*n* = 7)	Heavy	Asymmetrical	5 (71.4%)	2 (28.6%)	2 (28.6%)	5 (71.4%)
Total (*n* = 120)	Light (*n* = 54)	Symmetrical (*n* = 47)	82 (68.3%)	38 (31.7%)	53 (44.2%)	67 (55.8%)
	Heavy (*n* = 66)	Asymmetrical (*n* = 73)				

### Classification of playing posture

Specifically, playing posture was classified a priori based on whether the instrument was held symmetrically or asymmetrically relative to the midline of the body during a standard standing performance. Instruments held approximately along the sagittal midline without sustained trunk rotation or lateral flexion were classified as “symmetrical,” whereas instruments requiring deviation from the midline and potentially involving sustained trunk rotation or lateral inclination were classified as “asymmetrical.” The classification was determined by consensus among the authors based on direct observation of standard playing postures in the participating ensemble prior to data collection and was finalized prior to statistical analysis. Table 1 presents the classification of each instrument.

### Outcome measures

The present investigation employed an anonymous questionnaire with the following parameters: (1) experience of playing-related pain in the neck-and-shoulder region and low back, (2) pain severity while playing the instrument, and (3) duration of pain. Pain severity was defined as the pain intensity experienced specifically during instrument performance, and all pain-related questions were explicitly framed to assess symptoms occurring during performance. Pain severity was recorded using a numerical rating scale (NRS; 0 = no pain, 10 = worst imaginable pain; Additional file 1). Participants were asked to report the most severe pain experienced during instrument performance and the duration of that episode. If pain was experienced on multiple occasions, participants were instructed to indicate the episode with the greatest severity and its corresponding duration. This approach was intended to capture the maximum pain associated with instrument performance. Pain sites were classified a priori as “neck-and-shoulder region” and “low back.” A standardized body chart with predefined anatomical boundaries was not used. Consequently, participants reporting isolated neck pain and isolated shoulder pain were grouped within the same category. This approach may limit anatomical precision and should be interpreted accordingly. An NRS score of ≥ 4 was used to define moderate-to-severe pain (mild: 1–3, moderate: 4–6, severe: 7–10), consistent with its widespread use in pain research to represent at least moderate pain intensity associated with functional interference [13]. Pain duration of ≥ 3 months was used to indicate prolonged or chronic pain, based on the widely accepted definition of chronic pain established by the International Association for the Study of Pain [14].

This study was approved by the Institutional Review Board of the Kansai University of Welfare Sciences (approval no.: 18–29).

### Statistical analysis

The chi-square test was used to determine the possible relationships between the study parameters, playing posture, and instrument weight. The level of significance was set at *p* < 0.05. All statistical analyses were performed using IBM SPSS Statistics for Windows, version 24 (IBM Corp., Armonk, NY, USA).

## Results

When responses to specific severity or duration items were missing, percentages were calculated based only on participants who provided valid responses to the respective item. Table 1 presents the distribution of players by instrument type and the number of players who experienced neck-and-shoulder region pain and low-back pain. Overall, 82 (68.3%) students reported pain in the neck-and-shoulder region. The occurrence of pain in this region was not significantly associated with instrument weight or playing posture (Table 2). Moreover, 53 (44.2%) students experienced pain in the low back region; low-back pain was reported by a significantly higher proportion of students playing light instruments (30/54, 55.6%) than by those playing heavy instruments (23/66, 34.8%) (*p* < 0.05). However, no significant difference was observed between pain and playing posture (Table 3).

**Table 2:** Neck-and-shoulder region pain by instrument weight and playing posture

			Neck-and-shoulder region pain		
			Yes 82 (68.3%)	No 38 (31.7%)	χ^2^ *p* value
a) Instrument weight	Light (*n* = 54)		37 (68.5%)	17 (31.5%)	χ^2^ = 0.002 *p* = 0.969
	Heavy (*n* = 66)		45 (68.2%)	21 (31.8%)	
b) Playing posture	Symmetrical (*n* = 47)	30 (63.8%)		17 (36.2%)	χ^2^ = 0.724 *p* = 0.395
	Asymmetrical (*n* = 73)	52 (71.2%)		21 (28.8%)	

Data are presented as *n* (%) Of the 120 participants, 82 (68.3%) reported neck-and-shoulder region pain, whereas 38 (31.7%) did not

**Table 3:** Low-back pain by instrument weight and playing posture

			Low-back pain		
			Yes 53 (44.2%)	No 67 (55.8%)	χ^2^ *p* value
a) Instrument weight	Light (*n* = 54)		30 (55.6%)	24 (44.4%)	χ^2^ = 5.164 *p* = 0.023^*^
	Heavy (*n* = 66)		23 (34.8%)	43 (65.2%)	
b) Playing posture	Symmetrical (*n* = 47)	21 (44.7%)		26 (55.3%)	χ^2^ = 0.008 *p* = 0.927
	Asymmetrical (*n* = 73)	32 (43.8%)		41 (56.2%)	

Data are presented as *n* (%) Of the 120 participants, 53 (44.2%) reported low-back pain, whereas 67 (55.8%) did not. * *p* < 0.05.

Of the 82 students who experienced neck-and-shoulder region pain, 53 (64.6%) reported moderate-to-severe pain (NRS ≥ 4) while playing. Of these 82 students, 66 responded to the duration item, and 40 (60.6%) reported pain lasting ≥ 3 months. No significant relationship was observed between instrument weight or playing posture and these parameters (Tables 4 and 5).

**Table 4:** Neck-and-shoulder region pain severity by instrument weight and playing posture

		Moderate-to-severe neck-and-shoulder region pain (NRS ≥ 4)		
		Yes 53 (64.6%)	No 29 (35.4%)	χ^2^ *p* value
a) Instrument weight	Light (*n* = 37)	25 (67.6%)	12 (32.4%)	χ2 = 0.254 *p* = 0.614
	Heavy (*n* = 45)	28 (62.2%)	17 (37.8%)	
b) Playing posture	Symmetrical (*n* = 30)	18 (60.0%)	12 (40.0%)	χ2 = 0.444 *p* = 0.505
	Asymmetrical (*n* = 52)	35 (67.3%)	17 (32.7%)	

Data are presented as *n* (%) NRS: numerical rating scale Of the 82 participants who reported neck-and-shoulder region pain, 53 (64.6%) reported moderate-to-severe pain (NRS ≥ 4), whereas 29 (35.4%) reported mild pain (NRS < 4)

**Table 5:** Neck-and-shoulder region pain duration by instrument weight and playing posture

		Neck-and-shoulder region pain lasting ≥ 3 months		
		Yes 40 (60.6%)	No 26 (39.4%)	χ^2^ *p* value
a) Instrument weight	Light (*n* = 31)	18 (58.1%)	13 (41.9%)	χ2 = 0.158 *p* = 0.691
	Heavy (*n* = 35)	22 (62.9%)	13 (37.1%)	
b) Playing posture	Symmetrical (*n* = 25)	15 (60.0%)	10 (40.0%)	χ2 = 0.006 *p* = 0.937
	Asymmetrical (*n* = 41)	25 (61.0%)	16 (39.0%)	

Data are presented as *n* (%) Of the 82 participants who reported neck-and-shoulder region pain, 16 who did not respond to this item were excluded from the analysis. Among the remaining 66 participants, 40 (60.6%) reported pain lasting ≥ 3 months, whereas 26 (39.4%) reported pain lasting < 3 months

Of the 53 students who experienced low-back pain, 52 responded to the severity item, and 36 (69.2%) reported moderate-to-severe pain (NRS ≥ 4) while playing. A significantly greater proportion of participants with an asymmetrical playing posture (26/31, 83.9%) reported moderate-to-severe pain (NRS ≥ 4) than those with a symmetrical playing posture (10/21, 47.6%) (*p* < 0.01).

Of the 53 students who experienced low-back pain, 42 responded to the duration item, and 22 (52.4%) reported pain lasting ≥ 3 months. A significantly greater proportion of participants with an asymmetrical playing posture (17/26, 65.4%) reported prolonged pain than those with a symmetrical playing posture (5/16, 31.3%) (*p* < 0.05). No significant relationship was observed between instrument weight and these parameters (Tables 6 and 7).

**Table 6:** Low-back pain severity by instrument weight and playing posture

		Moderate-to-severe low-back pain (NRS ≥ 4)		
		Yes 36 (69.2%)	No 16 (30.8%)	χ^2^ *p* value
a) Instrument weight	Light (*n* = 30)	18 (60.0%)	12 (40.0%)	χ^2^ = 2.836 *p* = 0.092
	Heavy (*n* = 22)	18 (81.8%)	4 (18.2%)	
b) Playing posture	Symmetrical (*n* = 21)	10 (47.6%)	11 (52.4%)	χ^2^ = 7.724 *p* = 0.005^**^
	Asymmetrical (*n* = 31)	26 (83.9%)	5 (16.1%)	

Data are presented as *n* (%) NRS: numerical rating scale. ** *p* < 0.01. Of the 53 participants who reported low-back pain, one who did not respond to this item was excluded from the analysis. Among the remaining 52 participants, 36 (69.2%) reported moderate-to-severe pain (NRS ≥ 4), whereas 16 (30.8%) reported mild pain (NRS < 4).

**Table 7:** Low-back pain duration by instrument weight and playing posture

		Low-back pain lasting ≥ 3 months			
		Yes 22 (52.4%)		No 20 (47.6%)	χ^2^ *p* value
a) Instrument weight	Light (*n* = 22)	9 (40.9%)		13 (59.1%)	χ^2^ = 2.438 *p* = 0.118
	Heavy (*n* = 20)	13 (65.0%)		7 (35.0%)	
b) Playing posture	Symmetrical (*n* = 16)	5 (31.3%)	11 (68.8%)		χ^2^ = 4.627 *p* = 0.031^*^
	Asymmetrical (*n* = 26)	17 (65.4%)	9 (34.6%)		

Data are presented as *n* (%) * *p* < 0.05. Of the 53 participants who reported low-back pain, 11 who did not respond to this item were excluded from the analysis. Among the remaining 42 participants, 22 (52.4%) reported pain lasting ≥ 3 months, whereas 20 (47.6%) reported pain lasting < 3 months

## Discussion

In the present study, high school wind instrument players frequently reported pain in the neck-and-shoulder region, as well as the lower back. Moreover, many participants reported moderate-to-severe neck-and-shoulder and low-back pain (NRS ≥ 4) while playing a musical instrument, suggesting that playing may have increased the physical load on these areas. In addition, significantly more light-instrument players experienced low-back pain than heavy-instrument players. This finding warrants consideration of potential biomechanical mechanisms. Many light instruments are played held in front of the face and body, and therefore, the composite center of gravity position of the upper limbs and the instrument is considered to be deviated forward. Mitani et al. [12] measured spinal alignment while holding the playing posture of flute, trumpet, and marching euphonium in standing position and observed a decrease in the angle of trunk forward tilt compared with that in the resting standing position in the playing posture of these instruments. In addition, the muscle activity of the lumbar paraspinal muscle was most increased while playing the heavy instrument, marching euphonium. However, it was also increased while playing light instruments, like the flute and trumpet. This suggests that prolonged and/or intensive practice, which can lead to muscle fatigue, may also cause low-back pain. In contrast, heavy instruments such as the bass clarinet and tuba are often placed on a stand while playing. The bassoon is placed on a seat strap, whereas the euphonium is supported by the thighs. Therefore, playing such instruments appears to place minimal physical load on the low back. It is important to note that the marching euphonium, which produced the greatest increase in lumbar paraspinal muscle activity in our previous study [12], was not included in the present sample. The heavy instruments assessed here were predominantly those that were either structurally supported during the performance (e.g., tuba on a stand, bass clarinet with an endpin, bassoon with a seat strap, concert euphonium supported by the thighs) or positioned close to the body with minimal anterior trunk deviation (e.g., trombone, saxophones, French horn). These mechanical characteristics may reduce sustained lumbar loading compared to instruments that require unsupported forward holding. This distinction between supported and unsupported heavy instruments should be considered when interpreting our findings. Among heavy instruments that are not externally supported, the saxophone, French horn, and trombone are generally positioned close to the body. Although saxophone playing involves support from a neck strap and both hands, its proximity to the body and relatively low center of gravity may limit sustained anterior trunk deviation. The French horn is held close to the body with the bell directed laterally, although trombone playing, particularly during slide extension, may involve trunk rotation and lateral flexion. The instrument’s weight is primarily supported by the left hand and shoulder, with much of the mass positioned near the body’s center of mass. These characteristics may result in different lumbar loading patterns compared to light instruments that require sustained unsupported forward holding. Nevertheless, specific biomechanical demands may vary depending on the playing technique and repertoire. However, wind instrument players may perform in either a sitting or standing position. The difference in the physical load applied to the body while playing instruments in these positions is unclear and requires further investigation. These observations suggest that the concept of “functional load” or “postural load”—encompassing factors such as instrument mass, forward trunk inclination, asymmetrical trunk rotation, sustained static muscle activity, and overall body alignment—may be more relevant than instrument weight alone in explaining the development of low-back pain among wind instrument players. Future studies should incorporate direct biomechanical assessments of postural and functional loads to better characterize the physical demands imposed by different instruments. It should also be noted that all participants followed a standardized practice schedule of six days per week, approximately two hours per day on weekdays and six to eight hours per day on holidays. Although this relatively uniform exposure may have limited variability in practice frequency and daily duration, the participants varied in their instrumental playing history (ranging from less than one year to three or more years). Differences in the cumulative playing duration may influence both physical conditioning and musculoskeletal load. As playing history could not be incorporated as a covariate in the present analyses, its potential confounding effects should be considered when interpreting these findings.

Students who experienced low-back pain and played with an asymmetrical posture also reported greater pain severity and longer pain duration. From a biomechanical perspective, asymmetrical playing postures, such as those required for flute performance, involve sustained lateral flexion and axial rotation of the trunk relative to the pelvis [12]. These positions increase the eccentric demand on the contralateral paraspinal musculature and may generate asymmetrical compressive and shear forces on the lumbar intervertebral structures. Experimental biomechanical studies have demonstrated that combined loading patterns involving trunk flexion and rotation increase spinal tissue stress and may contribute to disc injury mechanisms [15]. Prolonged exposure to such loading patterns during practice may contribute to cumulative tissue stress and the development or exacerbation of low-back pain. An asymmetrical playing posture has previously been reported to increase the risk of musculoskeletal disorders [9], and the present findings are consistent with this.

Our results revealed no statistically significant association between instrument weight or playing posture and pain in the neck-and-shoulder region. This null finding may be explained by several factors. First, the overall prevalence of neck-and-shoulder pain was high (68.3%), suggesting a possible ceiling effect in which the near-universal exposure to playing-related cervical and shoulder demands may have obscured any differential effects of instrument weight or playing posture. Second, all players must maintain a sustained static arm position to hold their instrument, [16] recruit cervical and shoulder muscles to support embouchure formation during performance, [17] and in some cases, bear the additional load imposed by a neck strap. These ubiquitous demands may represent a baseline risk for neck-and-shoulder pain that affects players similarly across instrument categories, thereby potentially masking instrument-specific effects. Third, neck-and-shoulder region pain likely represents a heterogeneous symptom category involving multiple anatomical structures and mechanisms. The present study lacked anatomical specificity in the classification of neck-and-shoulder pain, as isolated neck pain and isolated shoulder pain were grouped within the same category. This aggregation may have reduced the sensitivity to detect differential associations with instrument characteristics. Fourth, the limited sample size of this exploratory study may have resulted in insufficient statistical power to detect true associations, and a Type II error cannot be excluded. Future studies with larger samples and anatomically specific pain classifications are warranted to clarify whether instrument weight and playing posture differentially affect neck and shoulder pain in wind instrument players. Accordingly, neck-and-shoulder pain in wind instrument players may be influenced more strongly by shared biomechanical demands than instrument-specific characteristics alone.

Based on the present findings, several practical implications are suggested for the prevention of PRMDs in adolescent wind instrument players. First, posture education should be incorporated into music training programs, with a particular emphasis on raising awareness of sustained trunk rotation and lateral flexion during performance. Students playing asymmetrical instruments, such as a flute, may especially benefit from regular postural assessments and guidance in maintaining a neutral spinal alignment where possible. Second, the incorporation of scheduled rest breaks during extended practice sessions is recommended to reduce cumulative musculoskeletal load, particularly for light instrument players who must sustain forward-held postures for prolonged periods. Third, preventive exercise programs targeting core muscle stabilization and paraspinal muscle strengthening may help enhance spinal load tolerance during sustained instrumental performance. Motor control–based exercise interventions have been effective in reducing chronic low-back pain in the general population [18]. Such findings suggest that similar preventive strategies may be beneficial for adolescent musicians, particularly those exposed to sustained asymmetrical postural demands. Early implementation of these strategies during high school may also help prevent the progression of playing-related pain into adulthood, given the known association between adolescent and adult low-back pain [10, 11].

This study has several limitations. First, as an exploratory cross-sectional investigation, causal relationships between instrument weight, playing posture, and musculoskeletal pain cannot be inferred. Several potential confounders, including playing experience, cumulative practice time, performance posture (sitting vs. standing), and individual physical characteristics, were not systematically adjusted for in the present analysis. Future studies employing longitudinal designs and multivariate analytical approaches are necessary to clarify the directionality and magnitude of these relationships while accounting for relevant confounding factors. Second, due to the limited sample size and distribution of instrument types, multivariate analyses using instrument weight and playing posture as explanatory variables could not be performed. In particular, the classification of instruments resulted in limited combinations, which restricted more detailed statistical modeling. Furthermore, although instrument playing history was collected and reported, it was not incorporated as a covariate into the analyses. This was partly because the study employed bivariate chi-square tests in an exploratory framework and the available sample size did not permit reliable multivariate modeling with additional covariates. In addition, daily practice schedules were relatively uniform across participants, reflecting the standardized brass band training structure, which may have reduced the variability in current playing exposure. Nevertheless, the cumulative playing duration may influence the development of musculoskeletal pain and could act as a potential confounder. Therefore, future studies with larger and more heterogeneous samples should incorporate playing history and cumulative exposure variables into multivariate analyses to clarify their roles in the development of playing-related musculoskeletal disorders. Moreover, the classification of instruments based solely on weight using a 1.0 kg cutoff was operational and not derived from established ergonomic criteria. The classifications were based on standard manufacturer-reported specifications rather than direct measurement of individual instruments, and the exact model and brand information was not recorded. Instrument weight alone may not fully reflect the biomechanical or functional load, which can also depend on posture, duration of static holding, and trunk rotation. Similarly, the playing posture classification was based on author consensus derived from direct observation and was not independently validated; formal inter-rater reliability assessment was not conducted. Therefore, both classifications should be regarded as heuristic operational categories rather than validated ergonomic measures, and the study findings should be interpreted cautiously. Future studies should consider treating instrument weight as a continuous variable and incorporating direct measures of functional or postural load to enable a more precise classification. Third, the study did not include a control group of general high school students. Musculoskeletal pain in adolescents is influenced by various daily activities, including the use of electronic devices and school-related physical demands, which may have affected our results. Therefore, the generalizability of our findings should be interpreted with caution. Fourth, the study sample consisted predominantly of female participants (107/120; 89.2%), reflecting the actual demographic composition of the brass band studied. Although this distribution was inherent to the study setting, potential sex-related differences in musculoskeletal pain were not examined. In addition, individual physical characteristics, including muscle strength, flexibility, and overall physical conditioning, were not objectively assessed, nor was physical activity outside instrumental practice. These factors may influence the spinal load tolerance and susceptibility to musculoskeletal pain during performance; however, their absence limits the comprehensiveness of the present assessment. Therefore, the findings should be interpreted with caution, particularly regarding the generalizability to male wind instrument players. Fifth, variables related to psychological well-being and academic performance were not assessed in the present study. These factors may be associated with playing posture and may influence the perception or reporting of musculoskeletal pain and could therefore act as potential confounders. Future studies adopting a broader biopsychosocial framework would provide a more comprehensive understanding of the determinants of playing-related musculoskeletal disorders in adolescent musicians. Sixth, pain severity and duration were assessed using retrospective self-reporting, and participants were asked to indicate the most severe pain experienced during instrument performance. This approach may be subject to recall bias, particularly when pain episodes occurred over an extended period. Although the questionnaire explicitly framed pain as playing-related, self-reported measures could not fully exclude the influence of non-playing-related factors. Notably, the use of the most severe pain episode rather than typical or average pain may have influenced the estimated prevalence of moderate-to-severe pain (NRS ≥ 4) and prolonged pain duration (≥ 3 months). Such responses may reflect transient or unusually severe episodes rather than habitual pain patterns during routine instrument performance. Consequently, the clinical implications from these findings should be interpreted cautiously. Future studies should consider prospective pain monitoring and the use of validated multidimensional questionnaires assessing both typical and peak pain intensity to enable more representative and clinically meaningful assessments. Seventh, this study did not distinguish between sitting and standing postures. Wind instrument players may perform in either position, and the differences in musculoskeletal loading between these postures remain unclear. Future studies should systematically consider performance posture as an additional analytical variable when examining playing-related musculoskeletal disorders. Finally, the pain site classification was based on self-reported responses without the use of a standardized body chart or predefined anatomical boundaries. The neck-and-shoulder region was treated as a combined site, which does not allow distinction between isolated neck and shoulder pain. Future studies should incorporate validated body charts to improve anatomical precision.

## Conclusions

This exploratory cross-sectional study investigated the onset of pain in the neck-and-shoulder region and low back among wind instrument players in a high school brass band and examined the relationships between pain, instrument weight, and playing posture. Accordingly, no significant relationship was observed between instrument weight or playing posture and pain in the neck-and-shoulder region, including pain severity and duration. Many participants who experienced low-back pain played light instruments. Moreover, an asymmetrical playing posture was associated with greater severity and longer duration of low-back pain.

## Supplementary Information

Supplementary Material 1.

## Data Availability

All data generated or analyzed during this study are included in this published article.

## Abbreviations

PRMDs: playing-related musculoskeletal disorders
NRS: numerical rating scale
