# Supplementary material for: Playing posture, instrument characteristics, and musculoskeletal pain among adolescent wind instrument players: an exploratory cross-sectional study
Source: BMC Musculoskelet Disord. 2026 Jun 5;27:673. doi: 10.1186/s12891-026-10061-2 (PMC13459380; doi:10.1186/s12891-026-10061-2)
Supplement: Supplementary file 1 [file 12891_2026_10061_MOESM1_ESM.docx]

**1. Participant Characteristics**

Sex:　☐ Male　　☐ Female

School year:　☐ First year　 　☐ Second year 　　☐ Third year

Age: (　　　　　　) years

Instruments played:

Which instrument(s) do you currently play? (	)

How long have you played this instrument? (	years	months)

How many days per week do you practice this instrument? (	days)

How many hours do you practice per day?
On weekdays: approximately (	) hours
On holidays: approximately (	) hours

**2. Pain in the Neck-and-Shoulder Region**

Have you experienced playing-related pain in the neck-and-shoulder region?

☐ Yes　　　 ☐ No

**If you answered “Yes,” please answer the following questions.**

Duration of pain: (	years	months)

***If you have experienced pain multiple times, please indicate the longest duration.**

Pain intensity while playing the instrument:

Please indicate the level of pain by circling the appropriate number below.

***If you have experienced pain multiple times, please indicate the most severe pain.**

0: No pain at all

10: Worst imaginable pain

**3. Pain in the Low Back**

Have you experienced playing-related pain in the low back?

☐ Yes　　　 ☐ No

**If you answered “Yes,” please answer the following questions.**

Duration of pain: (	years	months)

***If you have experienced pain multiple times, please indicate the longest duration.**

Pain intensity while playing the instrument:

Please indicate the level of pain by circling the appropriate number below.

***If you have experienced pain multiple times, please indicate the most severe pain.**

0: No pain at all

10: Worst imaginable pain
